# Psychological responses and cognitive mechanisms of university teachers in using generative AI in teaching: a configurational path analysis based on the MOA framework

**DOI:** 10.3389/fpsyg.2026.1798738

**Published:** 2026-06-12

**Authors:** Meiling Chen, Zhigang Xu, Shanying Li

**Affiliations:** 1 Academic Affairs Office, Longyan University, Longyan, China; 2 College of Resources and Environmental Engineering, Longyan University, Longyan, China; 3 College of Education, Longyan University, Longyan, China

**Keywords:** cognitive impacts, generative artificial intelligence, human-AI interaction, psychological responses, technical self-efficacy, university teachers

## Abstract

**Objective:**

The rapid integration of generative artificial intelligence (GenAI) into educational contexts has prompted significant attention to teachers' psychological responses and cognitive mechanisms as users of AI in teaching. While existing studies often focus on linear models that examine the net effects of single factors on technology acceptance, there is a lack of research exploring the complex psychological mechanisms behind teachers' GenAI use behaviors from a multi-factorial perspective. This study, based on the Motivation–Opportunity–Ability (MOA) framework, investigates the multi-path psychological drivers of university teachers' acceptance of GenAI in teaching.

**Methods:**

A survey was conducted with 258 teachers from Longyan University who have experience using GenAI in teaching. Using fuzzy-set qualitative comparative analysis (fsQCA), the study examined how motivational factors (hedonic motivation, performance expectancy), opportunity factors (social influence, facilitating conditions, and interactivity), and ability factors (AI literacy, technical self-efficacy) interact through different combinations to trigger high levels of GenAI acceptance in teaching.

**Results:**

The findings reveal that no single psychological or situational factor within the MOA framework independently explains high levels of GenAI adoption. Teachers' use of GenAI results from the synergistic interaction of multiple psychological responses and cognitive conditions. Six effective configurational paths were identified, categorized into three psychological models: performance expectancy-driven under technical self-efficacy, hedonic motivation-driven under ability support, and hedonic motivation-driven in high-interaction contexts.

**Conclusion:**

This study uncovers the multi-path psychological mechanisms behind university teachers' adoption of GenAI in teaching, expanding the explanatory power of the MOA framework in educational psychology and human-AI interaction research. The results offer empirical evidence for understanding teachers' psychological responses and decision-making processes in AI-supported teaching and provide theoretical insights for promoting healthy and effective AI usage behaviors among teachers.

## 1 Introduction

Since the launch of ChatGPT in 2022, generative artificial intelligence (GenAI) has rapidly advanced, evolving into multimodal and highly interactive applications (Wang and Li, 2024). Large language models, such as Baidu's “Wenxin Yiyan,” Alibaba's “Tongyi Qianwen,” and DeepSeek, have emerged, enabling AI to comprehend user intentions through complex algorithms and model structures, generating high-quality multimodal content, including text, images, and videos (Bai and Yang, 2025). In educational contexts, the application of GenAI is considered to have the potential to reshape teaching activities and learning methods, driving the digital transformation of education and having profound impacts on traditional teaching models, processes, and assessment methods (Wang et al., 2024).

With the widespread integration of GenAI into classroom teaching, an increasingly prominent issue in educational psychology has emerged: How do teachers, as users of artificial intelligence, form psychological responses and cognitive evaluations of GenAI in teaching contexts, and how do these responses influence their decisions regarding the adoption and continued use of the technology? university teachers are not only implementers of teaching innovation but also key players in determining whether GenAI can be effectively integrated into educational practices. Understanding teachers' real acceptance of GenAI in teaching and the psychological mechanisms driving their decisions is crucial for revealing the psychological foundations of human-AI interaction in education.

A growing body of recent research has examined teachers' acceptance of GenAI and related intelligent technologies, often based on theoretical frameworks such as the Technology Acceptance Model (TAM; Kong et al., 2024), the Unified Theory of Acceptance and Use of Technology (UTAUT; Qu et al., 2024), and the AI Device Use Acceptance Model (AIDUA; Zhang, 2025). These studies have provided valuable insights into the antecedents of teachers' adoption of intelligent technologies. However, most existing studies adopt variable-centered approaches such as structural equation modeling or regression analysis, which are particularly suitable for testing linear, net-effect, and theory-driven relationships among variables (Chen and Liu, 2020; Ragin and Fiss, 2008). In contrast, teachers' acceptance of GenAI in teaching may involve the joint influence of multiple motivational, contextual, and ability-related conditions, and different combinations of these conditions may lead to the same outcome. Therefore, a configurational approach can provide a useful complementary perspective for understanding the causal complexity underlying teachers' GenAI acceptance (Ragin and Fiss, 2008; Fiss, 2011).

Overall, the factors influencing teachers' acceptance of GenAI in teaching have yet to reach a consensus in the current research. On the one hand, there is variation in the psychological variables and situational factors explored across different studies. On the other hand, teachers' behaviors regarding GenAI usage are rarely driven by a single factor but rather emerge from the combined effects of various motivational, capability, and situational conditions. Few studies have explored how multiple psychological and situational conditions interact through different configurational paths to jointly shape teachers' behaviors in using GenAI for teaching.

In response to these gaps, this study adopts the Motivation–Opportunity–Ability (MOA) framework to examine university teachers' acceptance of GenAI in teaching from an integrated behavioral perspective (MacInnis and Jaworski, 1989; MacInnis et al., 1991). Compared with traditional technology acceptance frameworks that mainly emphasize the net effects of individual antecedents, the MOA framework highlights that behavior is jointly shaped by motivational drivers, contextual opportunities, and individual abilities (MacInnis et al., 1991). This perspective is particularly suitable for understanding teachers' GenAI use in teaching, because such behavior is unlikely to be driven by a single factor and may instead emerge from multiple combinations of psychological and situational conditions. Therefore, this study further employs fuzzy-set Qualitative Comparative Analysis (fsQCA), which is well-suited to capturing equifinality, conjunctural causation, and causal asymmetry, to uncover the multiple configurational pathways associated with high levels of GenAI acceptance in teaching (Ragin and Fiss, 2008; Fiss, 2011; Schneider and Wagemann, 2012).

## 2 Literature review

### 2.1 Theoretical foundation and research framework: AI usage behavior from the Motivation–Opportunity–Ability (MOA) perspective

The Motivation–Opportunity–Ability (MOA) theory was first proposed by (MacInnis and Jaworski 1989) in the context of marketing, to explain the psychological mechanisms involved in information processing and behavior selection. The theory posits that individual behavior is shaped not by a single factor but by the combined influence of three elements: motivation (the willingness to act), opportunity (the availability of situational conditions), and ability (the possession of relevant knowledge and skills; MacInnis and Jaworski, 1989; MacInnis et al., 1991). This theoretical framework emphasizes the interaction of multiple psychological and situational conditions, providing a valuable perspective for understanding complex behavioral decision-making processes.

As research on the MOA theory has expanded, it has been widely applied to various behavioral research domains, including user participation in knowledge communities (Huang and Yang, 2023), willingness to pay for academic platform knowledge (Wang, 2024), and user participation in online learning platforms (Huang, 2024), demonstrating strong explanatory power. Studies have shown that in the context of new technology adoption, individuals often need to comprehensively assess whether the technology can motivate usage, whether the necessary support conditions are in place, and whether they possess the necessary ability before making an adoption decision (Ahn and Chen, 2022; Xu et al., 2023).

**Figure 1:**
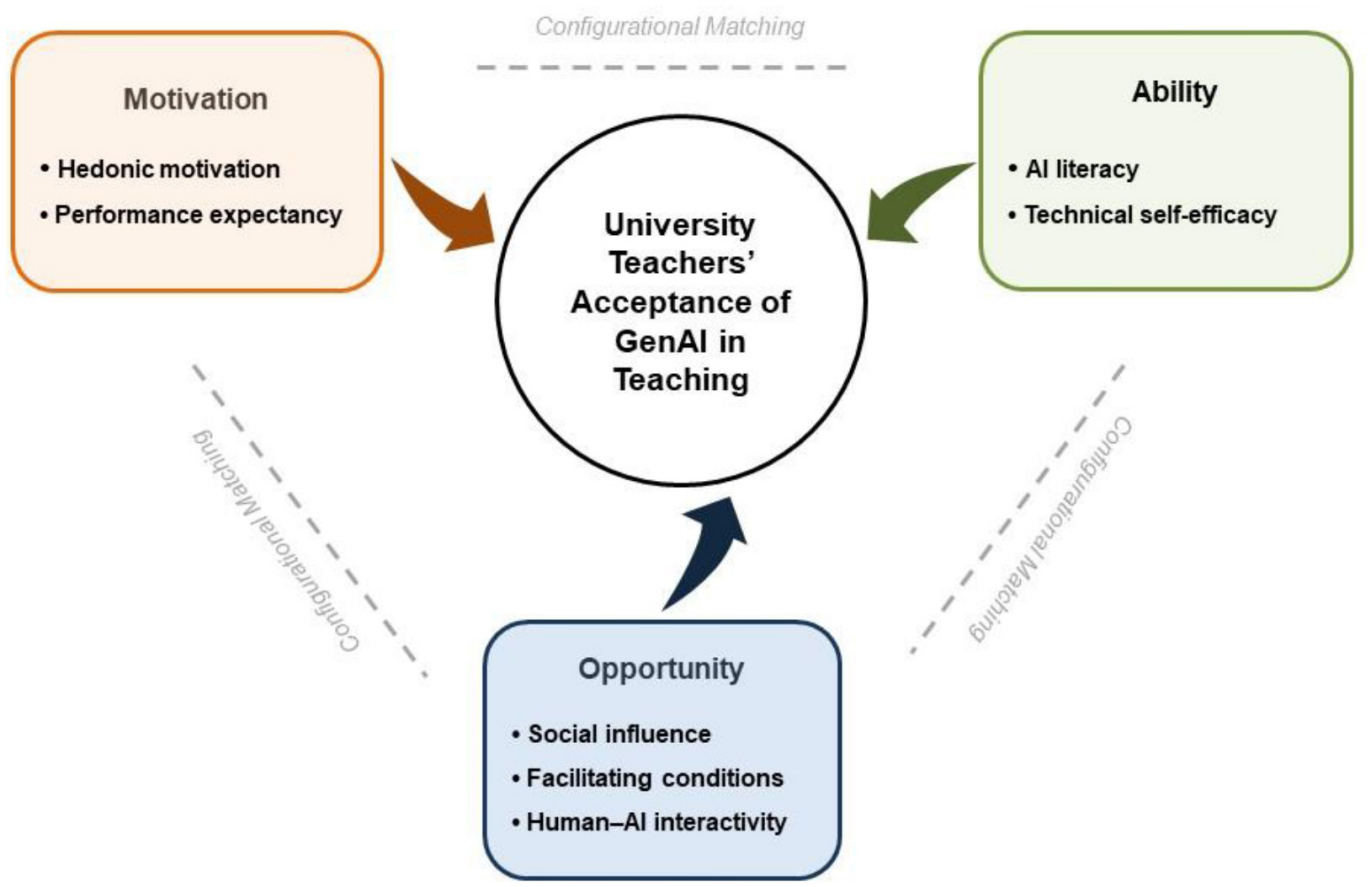
Research framework of university teachers' acceptance of GenAI in teaching based on the MOA framework.

In the context of AI application, whether teachers adopt and continue to use GenAI in teaching is not only influenced by their rational judgment of the technology's functionality but also by their psychological responses, cognitive evaluations, and situational perceptions. Therefore, the MOA theory provides an integrated analytical framework for understanding the psychological mechanisms of teachers as AI users. Based on this, the study constructs a research framework for university teachers' acceptance of GenAI in teaching, focusing on the three core dimensions of motivation, opportunity, and ability (see Figure 1), to reveal how various psychological and situational factors interact through different combinatory paths to shape teachers' GenAI adoption behaviors.

Existing studies have examined teachers' or users' acceptance of intelligent technologies based on frameworks such as the Technology Acceptance Model (TAM), the Unified Theory of Acceptance and Use of Technology (UTAUT), and the AI Device Use Acceptance Model (AIDUA), thereby enriching our understanding of the antecedents of technology acceptance. In general, TAM primarily emphasizes users' perceptions of usefulness and ease of use, UTAUT further incorporates performance expectancy, effort expectancy, social influence, and facilitating conditions, and AIDUA highlights the roles of cognitive appraisal and emotional responses in AI acceptance (Kong et al., 2024; Qu et al., 2024; Gursoy et al., 2019; Ma and Huo, 2023). These frameworks have provided important insights into the psychological antecedents of intelligent technology adoption. However, in the context of teachers' use of GenAI in teaching, acceptance is unlikely to depend on any single cognitive or emotional factor alone; rather, it may be shaped by the joint influence of motivational drivers, external support conditions, and individual capabilities. In particular, teachers' AI literacy and technical self-efficacy may directly affect how they understand, evaluate, and adopt GenAI in teaching practice. Therefore, compared with these prior models, the MOA framework provides a more suitable organizing structure for the present study because it integrates motivation, opportunity, and ability within a single behavioral framework and thus enables a more systematic examination of the multidimensional foundations of teachers' GenAI acceptance in educational settings (MacInnis and Jaworski, 1989; MacInnis et al., 1991; Ahn and Chen, 2022; Xu et al., 2023).

At the same time, this study does not assume that MOA exhausts all possible antecedents of GenAI acceptance. Recent research has also highlighted the roles of factors such as trust in AI, perceived risk, uncertainty, and AI-related anxiety. These factors are highly relevant to AI acceptance research (Choi et al., 2023; Shulner-Tal et al., 2023). However, to maintain a theoretically focused and parsimonious framework, the present study concentrates on key conditions that can be meaningfully organized within the motivation, opportunity, and ability dimensions. Future studies may further extend this framework by incorporating AI-specific factors into configurational analyses.

### 2.2 Motivation dimension: psychological drivers of teachers' GenAI adoption

In the MOA framework, motivation is considered the core psychological mechanism that initiates and sustains individual behavior (MacInnis et al., 1991). In educational contexts, teachers' technology adoption behaviors are often driven by their intrinsic interest, emotional experience, and expectations for achieving specific goals. The higher the motivation, the more likely an individual is to actively try and continue using new technology (Alhammadi et al., 2024).

Given the characteristics of GenAI and related research, this study characterizes the motivational structure underlying university teachers' adoption of GenAI in teaching from both intrinsic and instrumental motivation perspectives, with hedonic motivation and performance expectancy as the core variables of the motivation dimension.

#### 2.2.1 Hedonic motivation: intrinsic emotional experience in GenAI Use

Hedonic motivation refers to the pleasure, enjoyment, and emotional satisfaction individuals experience when using a particular technology (Chi et al., 2023), reflecting the intrinsic driving force behind technology use. In technology contexts with high interactivity and generative capabilities, hedonic motivation often becomes a key psychological factor that triggers technology usage.

Compared to traditional educational technologies that are primarily tool-driven, GenAI offers a more attractive user experience through its creative generation capabilities, personalized interactions, and real-time feedback mechanisms. Studies have shown that GenAI tools, such as ChatGPT, with their diverse modes of expression and human-like interaction features, significantly stimulate users' curiosity and willingness to use the technology (Bai and Yang, 2025). As a result, hedonic motivation is considered a significant psychological foundation influencing university teachers' adoption of GenAI in teaching.

#### 2.2.2 Performance expectancy: cognitive evaluation of GenAI's teaching value

Performance expectancy reflects an individual's cognitive assessment of the functional value of technology, specifically the expected impact of the technology on improving work performance, enhancing efficiency, or facilitating personal development (Ma and Huo, 2023; Bai and Yang, 2025). In the teaching context, university teachers' performance expectancy of GenAI primarily centers on whether it can optimize teaching effectiveness, reduce teaching workload, and support professional development.

Existing studies consistently indicate that when individuals perceive a new technology as highly useful, their willingness to adopt and actually use the technology increases (An et al., 2023; Ma, 2024; Guo, 2024). Therefore, performance expectancy, as a rational psychological response, plays a significant role in explaining teachers' acceptance of GenAI in teaching.

### 2.3 Opportunity dimension: contextual support conditions for GenAI in teaching

In the MOA theory, opportunity refers to the external environmental and situational conditions that facilitate a specific behavior (MacInnis and Jaworski, 1989). In this study, the opportunity dimension primarily focuses on the external support and situational constraints perceived by university teachers when adopting GenAI in teaching. Based on the context of AI educational applications, the study examines opportunity from three aspects: social influence, facilitating conditions, and interactivity.

#### 2.3.1 Social influence: normative pressure and professional expectations

Social influence is a widely recognized external factor in technology adoption research, referring to an individual's perception of the attitudes and expectations of significant others or the social environment regarding their technology use (Yilmaz et al., 2024). In educational contexts, school administrators, colleagues, educational policies, and industry development trends may all impact teachers' technology cognition and decision-making.

Research indicates that when teachers lack sufficient experience or information about new technology, the attitudes and role modeling of significant others play a particularly prominent role (Cheng et al., 2022; Sudirjo et al., 2023). Positive social evaluations and professional expectations help reduce teachers' perceptions of technological uncertainty, thereby enhancing their willingness to adopt (Li and Gu, 2021).

#### 2.3.2 Facilitating conditions: institutional and technological support environment

Facilitating conditions refer to the extent to which individuals perceive that organizational support, technological resources, and training conditions can ensure the use of technology (Venkatesh et al., 2003). In the university context, policy support, hardware and software facilities, and training opportunities are essential prerequisites for teachers to effectively use GenAI.

With the implementation of the Ministry of Education's “AI Innovation Action Plan for Higher Education Institutions,” creating a technology-enabled teaching environment has become a crucial direction for university development. Existing studies suggest a significant relationship between facilitating conditions and acceptance during the early stages of technology adoption (Jain et al., 2022; Wijaya et al., 2025). Therefore, incorporating facilitating conditions into the analysis provides a comprehensive understanding of the contextual foundations for teachers' GenAI adoption.

#### 2.3.3 Interactivity: human-AI interaction experience

Interactivity refers to the extent to which a GenAI system supports bidirectional dynamic communication in a teaching context, including factors such as feedback speed, response quality, and communication fluency (Liu et al., 2025). From a human-computer interaction perspective, high-quality interaction experiences enhance users' sense of control and trust, which further influences their technology adoption behavior.

Recent advances in AI systems' natural language understanding and generation have enabled them to adjust responses in real-time based on user needs, providing highly relevant and personalized information (Shulner-Tal et al., 2023; Gong, 2025). Research has indicated that a system's ability to provide high-quality feedback during interactions is a key factor in users' continued use of AI technologies (Lee and Oh, 2025).

### 2.4 Ability dimension: cognitive foundations and belief structures for teachers' use of GenAI

In the MOA theory, ability was initially defined as the knowledge, skills, and resources an individual possesses to carry out a specific behavior (MacInnis et al., 1991). Later research has further emphasized that ability includes not only objective skill levels but also individuals' subjective beliefs about their own capabilities (Wei et al., 2023; Yuan et al., 2025). In the context of AI educational applications, whether teachers possess sufficient ability directly influences their cognitive evaluations and usage behaviors regarding technology.

Based on related research, this study defines the ability dimension as the knowledge, skills, and confidence required by university teachers to effectively use GenAI in teaching, focusing on AI literacy and technical self-efficacy.

#### 2.4.1 AI literacy: understanding and evaluating AI's cognitive abilities

AI literacy refers to the comprehensive set of skills and knowledge that university teachers must possess to meet the educational demands of the intelligent era, covering teaching, research, and social service (Li et al., 2025). This literacy includes not only an understanding of the basic concepts and applications of AI but also the ability to critically assess its educational value and potential risks.

As AI continues to deeply integrate with teaching activities, AI literacy is viewed as a fundamental prerequisite for teachers to effectively integrate AI technology (Wang et al., 2023). Studies show that teachers with higher AI literacy are better able to identify AI's potential advantages in education, leading to more rational decisions about adoption and effective application (Kong et al., 2022; Zhao et al., 2022; Uygun, 2024).

#### 2.4.2 Technical self-efficacy: confidence and persistence in using GenAI

Self-efficacy refers to an individual's belief in their ability to complete a specific task (Bandura, 1994). In this study, technical self-efficacy refers to university teachers' confidence in their ability to adapt to and use GenAI for teaching activities (Choi et al., 2023; Masry-Herzallah and Watted, 2024).

Existing research highlights the key role of technical self-efficacy in shaping teachers' attitudes toward AI and their subsequent usage behaviors. Specifically, technical self-efficacy not only influences teachers' perceptions of AI's usability and limitations but also affects their willingness to use it and their persistence in overcoming technical challenges (Patil and Pramod, 2024; Herzallah and Makaldy, 2025). Thus, incorporating technical self-efficacy into the ability dimension helps to fully reveal the psychological foundations for university teachers' adoption of GenAI in teaching.

## 3 Methods

### 3.1 Research design and instrument development

This study adopted a survey methodology to systematically examine the psychological responses and related influencing factors of university teachers as users of GenAI in teaching contexts. To ensure that the participants had a basic understanding of the research topic, the survey questionnaire began with a brief explanation of the concept of generative AI tools and their typical applications. A screening question was included to ask participants whether they had practical experience using GenAI to assist in teaching. Only teachers with relevant usage experience were invited to complete the full questionnaire, ensuring the data's contextual relevance and validity.

The formal questionnaire consisted of three sections: The first section included a screening description and experience confirmation; the second section collected demographic information about the participants and their frequency of GenAI usage in teaching; the third section included measurement items for research variables, covering eight psychological and situational variables that influence university teachers' acceptance of GenAI in teaching. Each variable contained 3–4 measurement items.

The scale items were primarily adapted from widely validated mature scales used in domestic and international research, with appropriate revisions made to fit the specific context of university teachers' use of GenAI in teaching. During the scale development process, three experts in the relevant field were invited to evaluate the representativeness, clarity, and contextual suitability of the items. Based on their feedback, revisions were made to certain items, resulting in the final version of the measurement tool. Sample measurement items are shown in Table 1.

**Table 1:** Specific measurement items for each research variable.

Dimension	Research variable	Number of items	Sample items	References
Motivation	Hedonic motivation	3	HM1: Using GenAI tools for teaching is interesting and enjoyable.	Gursoy et al., 2019; Ma and Huo, 2023
			HM2: I enjoy exploring new features of GenAI tools.	
			HM3: Using GenAI tools enriches and makes my teaching experience more interesting.	
	Performance expectancy	4	PE1: Using GenAI helps me prepare for teaching (e.g., lesson preparation) more efficiently.	Venkatesh et al., 2012; Ma and Huo, 2023
			PE2: I expect that using GenAI will help me design more personalized teaching content to improve students' learning outcomes.	
			PE3: I expect that GenAI will provide innovative ideas for my teaching methods (e.g., intelligent interaction, adaptive learning).	
			PE4: I believe mastering GenAI tools will help enhance my professional competitiveness and career development.	
Opportunity	Social influence	4	SI1: Trends in the education field encourage me to adopt GenAI in teaching.	Strzelecki, 2024; Velli and Zafiropoulos, 2024
			SI2: School leaders place great emphasis on teachers' adoption of GenAI in teaching.	
			SI3: Some colleagues around me have already adopted GenAI for teaching, and I want to try it as well.	
			SI4: The concern that not using GenAI may make me fall behind educational technology trends motivates me to use it.	
	Facilitating conditions	4	FC1: The school has clear policy incentives that encourage teachers to develop smart courses, motivating me to use GenAI in teaching.	Strzelecki, 2024; Velli and Zafiropoulos, 2024
			FC2: The school organizes relevant technical training and experience sharing, which helps me use GenAI in teaching.	
			FC3: I can easily access the necessary GenAI teaching tools.	
			FC4: My teaching and research team or colleagues share GenAI teaching experiences and help each other, promoting the use of GenAI in teaching.	
	Interactivity	4	IN1: GenAI tools understand my instructions and respond accordingly in multi-turn conversations (e.g., generating course materials, designing questions, etc.).	Bai and Yang, 2025
			IN2: GenAI can adjust its interaction mode according to different teaching scenarios (e.g., lectures, group discussions, assignment guidance).	
			IN3: The GenAI teaching system provided by the school effectively supports real-time interaction among students, AI, and teachers (e.g., intelligent Q&A).	
			IN4: I can extract valuable student learning behavior analysis from interactions with GenAI.	
Ability	AI literacy	5	AL1: I can identify and correct issues in the content generated by GenAI tools (e.g., incorrect answers, unreasonable suggestions).	Al-Abdullatif, 2024
			AL2: I can avoid inputting sensitive student information (e.g., names, contact information) when using GenAI, following data privacy protection principles.	
			AL3: I can operate at least one GenAI tool (e.g., Deepseek, ChatGPT, Wenxin Yiyan) to complete specific teaching tasks (e.g., generating exercises, designing course outlines, class discussion questions, and case analysis).	
			AL4: I can use GenAI tools to design differentiated learning tasks for students at different levels (e.g., basic and advanced exercises).	
			AL5: I can use GenAI tools to access cutting-edge knowledge in the field and apply it to teaching improvements (e.g., the latest research trends, teaching cases).	
	Technical self-efficacy	3	TSE1: I believe I can learn to use GenAI tools for teaching.	Dong et al., 2020
			TSE2: I believe I can solve technical problems encountered when using GenAI (e.g., debugging prompts).	
			TSE3: I believe I can effectively integrate GenAI tools into my daily teaching activities.	
	Acceptance	3	ACC1: I am willing to use GenAI tools in future teaching.	Al-Abdullatif, 2024
			ACC2: I am willing to recommend GenAI tools to other teachers.	
			ACC3: I plan to incorporate GenAI into my teaching activities (e.g., instructional design, classroom implementation, assessment feedback).	

All measurement items were assessed using a five-point Likert scale, ranging from 1 (“Strongly Disagree”) to 5 (“Strongly Agree”), where participants selected responses based on their actual experiences and subjective perceptions.

### 3.2 Participants and data collection

This study focused on full-time teachers at Longyan University and collected data through an online survey. A total of 329 questionnaires were initially returned. To ensure data quality, the sample was screened in several steps. First, 38 questionnaires from respondents without practical experience using GenAI in teaching were excluded. Second, questionnaires showing uniform response patterns were not excluded solely on that basis; rather, they were removed only when such patterns were accompanied by very short completion time (less than 1 min) and/or logical inconsistencies across related items. Based on this combined assessment, 33 questionnaires were excluded. Finally, 258 valid questionnaires were retained for subsequent analysis, yielding an effective response rate of 78.42% (see Table 2). This sample size is adequate for conducting fsQCA-based configurational analysis.

**Table 2:** Survey sample characteristics.

Variable	Category	Number	Percentage (%)
Gender	Male	124	48.0
	Female	134	52.0
Teaching experience	0–5 years	44	17.1
	6–10 years	26	16.5
	11–20 years	117	45.3
	21 years and above	71	21.1
Academic title	Assistant	17	6.6
	Lecturer	77	29.8
	Associate Professor	128	50.4
	Professor	36	13.2
Subject area	Humanities and Social Sciences	134	51.9
	Science and Engineering	124	48.1
Frequency of GenAI teaching use	Daily	65	25.2
	Once a week or more	110	42.6
	Occasionally	83	32.2

### 3.3 Reliability and validity assessment

This study first used SPSS to conduct descriptive statistical analysis and preliminary data management. SmartPLS was then used to assess the measurement model, because the dimensions and constructs in this study were theoretically predefined based on the MOA framework and adapted from established scales in prior research. In this sense, the two tools served different but complementary purposes: SPSS was used for descriptive analysis, whereas SmartPLS was used for reliability and construct-validity assessment.

As shown in Table 3, the Cronbach's alpha values of all constructs exceeded the recommended threshold of 0.70, indicating satisfactory internal consistency reliability. In addition, all factor loadings were above 0.70, the composite reliability (CR) values were above 0.80, and the average variance extracted (AVE) values were above 0.60, indicating good convergent validity (Fornell and Larcker, 1981). Discriminant validity was assessed using the Fornell–Larcker criterion. As shown in Table 4, the square root of the AVE for each construct was greater than its correlations with other constructs, supporting satisfactory discriminant validity.

**Table 3:** Reliability and convergent validity analysis of the scale.

Research variable	Measurement items	Factor loadings	Cronbach's α	Composite reliability (CR)	Average variance extracted (AVE)
Hedonic Motivation (HM)	HM1	0.861	0.857	0.886	0.777
	HM2	0.899			
	HM3	0.884			
Performance Expectancy (PE)	PE1	0.849	0.911	0.928	0.789
	PE2	0.905			
	PE3	0.908			
	PE4	0.891			
Social Influence (SI)	SI1	0.903	0.854	0.893	0.696
	SI2	0.706			
	SI3	0.889			
	SI4	0.823			
Facilitating Conditions (FC)	FC1	0.815	0.838	0.871	0.672
	FC2	0.840			
	FC3	0.826			
	FC4	0.797			
Interactivity (IN)	IN1	0.860	0.906	0.922	0.781
	IN2	0.930			
	IN3	0.860			
	IN4	0.883			
AI Literacy (AL)	AL1	0.835	0.883	0.902	0.681
	AL2	0.776			
	AL3	0.849			
	AL4	0.807			
	AL5	0.857			
Technical Self-Efficacy (TSE)	SE1	0.915	0.897	0.920	0.829
	SE2	0.879			
	SE3	0.936			
Acceptance (ACC)	ACC1	0.923	0.870	0.898	0.794
	ACC 2	0.906			
	ACC 3	0.843			

**Table 4:** Discriminant validity analysis of the scale.

Variable	AL	FC	HM	IN	PE	TSE	SI	ACC
AL	0.825							
FC	0.761	**0.820**						
HM	0.607	0.625	**0.882**					
IN	0.742	0.745	0.556	**0.884**				
PE	0.715	0.701	0.782	0.693	**0.888**			
TSE	0.769	0.672	0.558	0.662	0.622	**0.911**		
SI	0.747	0.787	0.672	0.738	0.823	0.682	**0.834**	
ACC	0.700	0.635	0.578	0.695	0.684	0.833	0.742	**0.891**

Bold values on the diagonal represent the square roots of the average variance extracted (AVE) for each construct; off-diagonal values represent the correlations between constructs.

### 3.4 Rationale for using fsQCA

This study adopts fuzzy-set Qualitative Comparative Analysis (fsQCA) to examine the configurational effects of motivation, opportunity, and ability conditions on university teachers' acceptance of GenAI in teaching (Ragin and Fiss, 2008; Fiss, 2011). The choice of fsQCA is closely related to the theoretical premise of this study. Under the MOA framework, teachers' acceptance of GenAI is assumed to be jointly shaped by multiple dimensions rather than determined by a single antecedent. Different combinations of motivational, contextual, and ability-related conditions may lead to the same outcome, which reflects equifinality (MacInnis et al., 1991; Ragin and Fiss, 2008). In addition, the effect of one condition may depend on the presence or absence of other conditions, which reflects conjunctural causation. Moreover, the causal pathways leading to high acceptance may not simply mirror those leading to its absence, which reflects causal asymmetry (Ragin and Fiss, 2008; Fiss, 2011; Schneider and Wagemann, 2012). Because fsQCA is well-suited to capturing these features of causal complexity, it provides a useful complementary approach to variable-centered methods such as regression and structural equation modeling, which are more appropriate for testing linear and net-effect relationships among variables (Ragin and Fiss, 2008; Fiss, 2011).

### 3.5 Calibration and fsQCA procedure

Before conducting the fsQCA analysis, the original scale data were calibrated into fuzzy-set membership scores. First, the arithmetic mean of the items measuring each construct was calculated to obtain a composite score. Following Ragin's direct calibration approach, and given the absence of externally established substantive cutoffs, this study adopted an empirical percentile-based calibration scheme (Ragin and Fiss, 2008). Specifically, the 95th percentile, 50th percentile, and 5th percentile were used as the thresholds for full membership, crossover, and full non-membership, respectively (Ragin and Fiss, 2008). The calibrate function in fsQCA 3.0 was then used to transform the original continuous scores into fuzzy-set membership scores ranging from 0 to 1. To avoid the automatic exclusion of cases with a membership score exactly equal to 0.5 in the truth-table algorithm, all such values were slightly adjusted to 0.501 (Cai and Yang, 2022). Table 5 presents the calibration criteria and an illustrative example.

**Table 5:** Calibration anchors and illustrative case for direct calibration.

Dimension	Condition/outcome	Full non-membership (5th percentile)	Crossover (50th percentile)	Full membership (95th percentile)	Scale range	Illustrative raw score → fuzzy membership
**Motivation**	Hedonic motivation (HM)	2.98	4.00	5.00	1–5	4.00 → 0.501
	Performance expectancy (PE)	2.75	4.00	5.00	1–5	3.75 → 0.35
**Opportunity**	Social Influence (SI)	3.00	4.00	5.00	1–5	3.00 → 0.05
	Facilitating Conditions (FC)	2.75	4.00	5.00	1–5	3.25 → 0.14
	Interactivity (IN)	2.50	4.00	5.00	1–5	3.75 → 0.38
**Ability**	AI Literacy (AL)	3.00	4.00	5.00	1–5	3.20 → 0.08
	Technical Self-Efficacy (TSE)	3.00	4.00	5.00	1–5	3.33 → 0.12
**Outcome**	Acceptance (ACC)	3.00	4.00	5.00	1–5	3.67 → 0.27

All anchors were derived using the direct calibration method based on the 5th, 50th, and 95th percentiles of the sample distribution. Because all constructs were measured on a five-point Likert scale, the theoretical upper bound of each variable is 5. In the present sample, the 95th percentile for all conditions and the outcome reached 5.00; therefore, the full-membership anchor was uniformly set at 5.00. This does not violate the percentile-based calibration principle. Similarly, the median of each calibrated set was 4.00, so the crossover anchor was uniformly set at 4.00. By contrast, the full non-membership anchors vary across conditions because they reflect differences in the lower-tail distributions of the respective variables. The illustrative case is provided only to demonstrate the calibration procedure and does not represent a specific threshold or boundary case. To avoid being excluded due to exact membership scores of 0.500 in the truth-table procedure, such cases were adjusted to 0.501.

After calibration, a necessity analysis was conducted to examine whether any single antecedent condition constituted a necessary condition for high GenAI acceptance in teaching. Following common fsQCA practice, a consistency threshold of 0.90 was used to assess necessity (Schneider and Wagemann, 2012).

For the sufficiency analysis, a truth table was constructed using fsQCA 3.0. Following commonly used standards in prior research, the consistency threshold was set at 0.80, the case frequency threshold was set at 1, and the proportional reduction in inconsistency (PRI) threshold was set at 0.70 (Fiss, 2011; Schneider and Wagemann, 2012). Intermediate solutions were used for interpretation, and conditions appearing in both the parsimonious and intermediate solutions were treated as core conditions, whereas those appearing only in the intermediate solution were treated as peripheral conditions (Fiss, 2011).

## 4 Results

University teachers' high acceptance of GenAI in teaching is associated with the combined effects of multiple motivation-, opportunity-, and ability-related conditions rather than any single psychological or situational factor. Based on the fsQCA results, this section reports the empirical findings regarding necessary conditions, configurational pathways, and robustness.

### 4.1 Necessary condition analysis

The necessity analysis showed that the consistency values of all antecedent conditions and their negations were below 0.90, indicating that no single condition constituted a necessary condition for high GenAI acceptance in teaching. The results of the necessary condition analysis and configurational path analysis are presented in Tables 6 and 7, respectively.

**Table 6:** Univariate necessity analysis for high GenAI teaching acceptance.

Dimension	Variable	Condition	Consistency	Coverage	Meets necessity criterion (Consistency ≥0.90)
**Motivation**	Hedonic motivation	Present (HM)	0.74	0.88	No
		Absent (~HM)	0.67	0.59	No
	Performance expectancy	Present (PE)	0.85	0.86	No
		Absent (~PE)	0.62	0.62	No
**Opportunity**	Social influence	Present (SI)	0.86	0.87	No
		Absent (~SI)	0.63	0.64	No
	Facilitating conditions	Present (FC)	0.74	0.89	No
		Absent (~FC)	0.71	0.62	No
	Interactivity	Present (IN)	0.81	0.89	No
		Absent (~IN)	0.66	0.61	No
**Ability**	AI literacy	Present (AL)	0.77	0.89	No
		Absent (~AL)	0.68	0.61	No
	Technical self-efficacy	Present (TSE)	0.86	0.93	No
		Absent (~TSE)	0.65	0.62	No

Antecedent conditions are grouped by MOA dimension. “Present” and “Absent” indicate the presence and negation of each condition, respectively. All consistency values are below the conventional threshold of 0.90, indicating that no single antecedent condition constitutes a necessary condition for high GenAI teaching acceptance. Among the listed conditions, technical self-efficacy and social influence show relatively higher consistency, but neither reaches the threshold for necessity.

**Table 7:** Configurational analysis for high GenAI teaching acceptance.

Dimension	Condition	C1a	C1b	C2a	C2b	C2c	C3
Motivation	Hedonic motivation	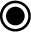		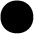	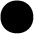	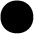	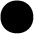
	Performance expectancy	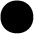	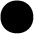	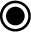		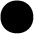	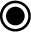
Opportunity	Social influence	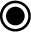	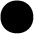	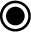	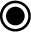		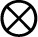
	Facilitating conditions	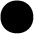			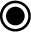	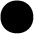	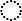
	Interactivity		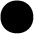		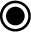		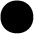
Ability	AI literacy		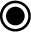	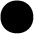	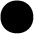	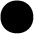	
	Technical self-efficacy	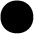	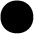	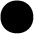	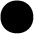	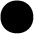	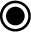
Raw coverage		0.56	0.60	0.58	0.52	0.54	0.34
Unique coverage		0.01	0.07	0.02	0.01	0.00	0.01
Consistency		0.97	0.97	0.97	0.97	0.97	0.97
Overall coverage		0.71					
Overall consistency		0.96					

In line with standard fsQCA reporting conventions, 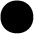 indicates the presence of a core condition, 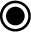 indicates the presence of a peripheral condition, 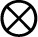 indicates the absence of a core condition, 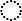 indicates the absence of a peripheral condition, and a blank cell indicates that the presence or absence of the condition is not relevant to that specific configuration. Following the comparison between the parsimonious and intermediate solutions, conditions appearing in both solutions are treated as core conditions, whereas those appearing only in the intermediate solution are treated as peripheral conditions. The six configurations are grouped according to their dominant theoretical logic: C1a and C1b represent the performance expectancy-driven model supported by technical self-efficacy, C2a, C2b, and C2c represent the hedonic motivation-driven model under ability support, and C3 represents the hedonic motivation model in high-interaction contexts. To improve readability, the table presents configurational patterns first, followed by the solution statistics for each configuration. Among the six solutions, C1b shows the highest raw coverage, indicating comparatively greater empirical relevance in the observed sample, whereas all six configurations exhibit high consistency (0.97). Overall, the solution coverage is 0.71 and the overall solution consistency is 0.96, indicating satisfactory explanatory power and internal consistency of the configurational solution.

### 4.2 Configurational paths to high GenAI acceptance

Since no single condition constituted a necessary condition, the configurational analysis was conducted to identify combinations of antecedent conditions associated with high levels of GenAI acceptance in teaching. Six configurations were identified. The overall solution coverage was 0.71 and the overall solution consistency was 0.96, indicating satisfactory explanatory relevance and consistency. Across these configurations, motivation, opportunity, and ability conditions jointly contributed to high GenAI acceptance, further supporting the multidimensional psychological basis of teachers' GenAI adoption behavior.

Based on the core condition structures, the six configurational paths were categorized into three types.

#### 4.2.1 Performance expectancy-driven model supported by technical self-efficacy

This model includes two paths: C1a and C1b. Both paths feature performance expectancy (PE) and technical self-efficacy (TSE) as core conditions, indicating that teachers' rational evaluation of GenAI's teaching value and their confidence in their technical abilities are key psychological foundations for triggering high acceptance.

In the C1a path, performance expectancy and technical self-efficacy are core conditions, with facilitating conditions (FC) also forming a core condition, and hedonic motivation (HM) and social influence (SI) as auxiliary conditions. This path shows that when teachers possess strong technical self-efficacy, perceive GenAI's performance benefits, and receive sufficient institutional and resource support, high adoption behavior can occur, even if enjoyment and social pressure only play auxiliary roles.

The C1b path also includes performance expectancy and technical self-efficacy as core conditions, with social influence (SI) and interactivity (IN) as core conditions, and AI literacy (AL) as an auxiliary condition. This path shows that in strong social norms and high-quality human-AI interaction contexts, teachers' functional knowledge and ability beliefs can be further reinforced.

#### 4.2.2 Hedonic motivation-driven model under ability support

This model includes three paths: C2a, C2b, and C2c, all of which feature AI literacy (AL), technical self-efficacy (TSE), and hedonic motivation (HM) as core conditions, highlighting the key role of ability and intrinsic emotional experience in teachers' GenAI adoption.

In the C2a path, the three core conditions are combined with performance expectancy (PE) and social influence (SI) as auxiliary conditions. The result indicates that when teachers possess strong AI literacy and technical confidence and experience enjoyment during use, high adoption willingness can be formed even when perceptions of performance benefits and social support are not dominant.

In the C2b path, the three core conditions are combined with social influence (SI), facilitating conditions (FC), and interactivity (IN) as auxiliary conditions, reflecting that in multiple support contexts, the combination of ability and hedonic motivation is more likely to lead to stable adoption behavior.

In the C2c path, performance expectancy (PE) and facilitating conditions (FC) rise from auxiliary conditions to core conditions, forming a driving structure with ability and hedonic motivation. This path shows that when teachers possess both ability and emotional motivation, and perceive GenAI's performance value with adequate support, their adoption behavior becomes more certain.

#### 4.2.3 Hedonic motivation model in high-interaction contexts

The C3 path centers on hedonic motivation (HM) and interactivity (IN) as core conditions, with performance expectancy (PE) and technical self-efficacy (TSE) as auxiliary conditions. This path shows that, even in the absence of prominent social influence and facilitating conditions, teachers can still form high adoption behavior if they receive high-quality responses and positive emotional experiences during interactions with GenAI, while possessing basic technical confidence and performance expectations. This highlights the independent role of the human-AI interaction experience in driving teachers' adoption behavior in specific contexts.

### 4.3 Robustness check

To assess the robustness of the configurational findings, a supplementary analysis was conducted using stricter truth-table settings. Following prior fsQCA studies, the consistency threshold was increased from 0.80 to 0.85 and the case frequency threshold was raised from 1 to 2, while the other settings remained unchanged (Fiss, 2011; Schneider and Wagemann, 2012).

## 5 Discussion

The results of this study indicate that university teachers' high-level acceptance of GenAI in teaching is primarily driven by three distinct psychological models: the performance expectancy-driven model supported by technical self-efficacy, the hedonic motivation-driven model under ability support, and the hedonic motivation model in interactive contexts. These three models reflect different combinations of rational evaluations, emotional experiences, and beliefs in ability when teachers engage with GenAI.

### 5.1 Performance expectancy-driven model supported by technical self-efficacy

In this model, technical self-efficacy and performance expectancy serve as core conditions, forming the primary psychological basis for teachers' adoption of GenAI in teaching. The results show that when university teachers possess high technical self-efficacy, meaning they believe in their ability to effectively handle the potential technological challenges in using GenAI, and when they clearly perceive the practical value of GenAI in enhancing teaching effectiveness, optimizing teaching processes, or promoting professional development, they are more likely to exhibit high levels of acceptance.

This finding aligns closely with the path of “efficacy beliefs—effort investment—performance expectations” in social cognitive theory. Previous research has shown that individuals with high technical self-efficacy are more likely to invest effort in new technology use and continuously improve their skills through active engagement (Xie et al., 2022). This increased sense of capability further strengthens their positive expectations about the technology's performance, facilitating technology adoption (Altalhi, 2021; Wang et al., 2024). In the context of GenAI teaching applications, technical self-efficacy not only reduces teachers' perception of uncertainty but also provides psychological support for forming performance expectations.

### 5.2 Hedonic motivation-driven model under ability support

The second model centers on AI literacy, technical self-efficacy, and hedonic motivation, emphasizing the synergistic effect of ability and intrinsic emotional experience. The results indicate that when university teachers possess high AI literacy, understand the basic principles, potential, and limitations of GenAI, and have strong confidence in their ability to use GenAI for teaching, experiencing enjoyment and pleasure during use is sufficient to trigger high-level acceptance of GenAI in teaching.

This finding supports the crucial role of AI literacy in reducing technological uncertainty. Previous research has shown that higher AI literacy helps individuals more accurately identify suitable use cases for technology, reducing hesitation or misunderstanding due to cognitive ambiguity (Long and Magerko, 2020; Laupichler et al., 2022). When this cognitive clarity is combined with positive emotional experiences, teachers are more likely to view GenAI as a valuable and sustainable teaching tool (Ng et al., 2021; Wang et al., 2023). Therefore, this model reveals that, under conditions of adequate ability, hedonic motivation can serve as a key psychological trigger for driving teachers' adoption of GenAI.

### 5.3 Hedonic motivation model in interactive contexts

The third model focuses on interactivity and hedonic motivation as core conditions, presenting a psychology-driven pathway centered on the user experience. The study found that in contexts where social influence and facilitating conditions are relatively weak, university teachers' pay more attention to the quality of the interaction with GenAI itself. When GenAI is able to accurately understand commands, provide quick feedback, and offer targeted responses, this high-quality human-AI interaction experience can directly evoke positive emotional reactions from teachers, thereby promoting their adoption behavior.

This finding highlights the bridging role of interactivity in connecting technological capability with user psychological responses. Previous studies have pointed out that emotional experiences, such as fun, pleasure, or immersion, during interactions with GenAI directly modulate users' attitudes and willingness to use the technology (Bai and Yang, 2025). This study further suggests that, even with limited external support conditions, teachers can still form high-level adoption intentions as long as the interaction experience is sufficiently positive, emphasizing the independent value of human-AI interaction quality in educational contexts.

### 5.4 The central role of technical self-efficacy across multiple paths

The results show that technical self-efficacy plays a key role in all six configurational paths, with five paths featuring it as a core condition and one path as an auxiliary condition. This indicates that technical self-efficacy is central to nearly all psychological paths that lead to high-level GenAI acceptance in teaching by university teachers.

This finding further confirms the foundational status of technical self-efficacy in AI educational applications. Previous research has pointed out that technical self-efficacy not only influences teachers' attitudes toward AI technology but also affects the effort and persistence they invest in practice (Masry Herzallah and Makaldy, 2025). In the context of GenAI teaching applications, technical self-efficacy reduces technology anxiety by enhancing teachers' sense of control and confidence, providing the necessary psychological foundation for the activation of various motivational and situational conditions.

### 5.5 The complementary driving effects of hedonic motivation and performance expectancy

Further analysis reveals that hedonic motivation and performance expectancy appear as core conditions in at least one form across all configurational paths. Two paths center on performance expectancy, three paths center on hedonic motivation, and one path depends on both. This result suggests that the psychological drivers behind teachers' adoption of GenAI in teaching are not singular but exhibit characteristics of both instrumental value and emotional experience.

This finding expands the perspective of previous research, which predominantly focused on rational decision-making in technology adoption. Teachers may adopt GenAI due to the performance improvements it brings to teaching, but they may also form a sustained intention to use it because of the enjoyment experienced during use. Both factors play complementary roles in different psychological pathways, together forming a complex decision-making foundation for university teachers as users of AI.

At the same time, these configurational findings should be interpreted with caution. The identified pathways should not be understood as universal or exclusive explanations of teachers' GenAI acceptance, but rather as context-bound configurational patterns observed in the present sample. Different teachers may rely on different combinations of motivational, contextual, and ability-related conditions, and the relative salience of these conditions may vary across institutional settings. In addition, although this study focuses on MOA-based conditions, other AI-related factors such as trust, perceived risk, uncertainty, and AI-related anxiety may also shape teachers' acceptance of GenAI (Choi et al., 2023; Shulner-Tal et al., 2023). Future research may extend the present framework by incorporating these factors into broader configurational analyses.

## 6 Conclusion

With the rapid integration of GenAI into educational contexts, the psychological responses and cognitive mechanisms of teachers, as AI users, have become an important research topic in educational psychology. Based on the Motivation–Opportunity–Ability (MOA) theoretical framework, this study employs fuzzy-set Qualitative Comparative Analysis (fsQCA) to systematically explore the multiple pathways to high-level acceptance of GenAI in teaching by university teachers from a configurational perspective.

The results indicate that university teachers' high acceptance of GenAI in teaching is associated not with any single psychological or situational factor, but with different combinations of motivational, ability-related, and contextual conditions. Specifically, this study identifies three representative configurational models: a performance expectancy-centered model supported by technical self-efficacy, an hedonic motivation-centered model under ability support, and an hedonic motivation-centered model in high-interaction contexts. These configurations help explain how rational value judgments, emotional experiences, and ability beliefs may work together in different ways in shaping teachers' acceptance of GenAI in teaching.

From a theoretical perspective, this study extends the explanatory boundaries of the MOA framework in educational psychology and AI in education research (MacInnis and Jaworski, 1989; MacInnis et al., 1991). Compared to traditional linear model-based technology adoption studies, this research reveals the mechanisms of the concurrent effects of multiple psychological conditions through configurational analysis, emphasizing the differentiated roles of technical self-efficacy, hedonic motivation, and performance expectancy across different paths (Ragin and Fiss, 2008; Fiss, 2011). This finding provides a more nuanced and dynamic perspective for understanding the psychological responses and cognitive impacts of AI users.

From a practical perspective, the findings offer targeted insights for promoting the use of GenAI in teaching within universities. On one hand, efforts should be made to enhance teachers' technical self-efficacy and AI literacy through training and support mechanisms, providing a foundation for positive technology perceptions. On the other hand, the interactive experience and emotional feedback in the use of GenAI tools should be emphasized, ensuring that teachers experience positive and enjoyable interactions during teaching practice. Additionally, universities could implement differentiated support strategies tailored to the psychological driving models of different teacher groups to promote the rational, effective, and sustainable application of GenAI in teaching.

Overall, this study reveals, from an educational psychology perspective, the multi-path psychological mechanisms of university teachers as GenAI users in teaching contexts, providing empirical evidence for understanding the psychological impact of AI on educational practice. Future research could further validate and extend the findings of this study in different educational levels and cultural contexts to deepen the understanding of the long-term psychological effects of human-AI interaction in education.

## 7 Limitations

This study has several limitations. First, the sample was drawn from a single university, which may limit the generalizability of the findings to other institutional or cultural contexts. Second, the data were collected through a cross-sectional self-report survey, which limits strong causal interpretation and may still involve common method concerns despite the procedural and statistical precautions taken. Third, the present study focused on MOA-based conditions and did not include other potentially relevant AI-specific factors such as trust in AI, perceived risk, uncertainty, or AI-related anxiety. Future research could examine whether these factors form additional configurations across more diverse samples and educational settings.

## 8 Policy recommendations

Based on the findings of this study regarding the psychological mechanisms underlying university teachers' high-level acceptance of GenAI in teaching, several policy recommendations can be made at three levels: empowering teachers' abilities, motivating engagement, and optimizing the human-AI collaborative environment, all aimed at supporting university teachers to more effectively and sustainably adopt GenAI-assisted teaching.

### 8.1 Implement GenAI empowerment initiatives to systematically enhance teachers' teaching capabilities

The study results indicate that technical self-efficacy and AI literacy play a core role in multiple configurational paths, serving as the key psychological foundations for high-level acceptance of GenAI in teaching. This finding suggests that universities should implement systematic empowerment measures to enhance teachers' understanding and confidence in using GenAI. Previous research has also highlighted that targeted technical training and support can significantly improve individuals' willingness to adopt new technologies (Elnadi and Gheith, 2021).

Therefore, it is recommended that universities implement structured GenAI teaching empowerment initiatives. First, universities could regularly organize GenAI training sessions for all full-time faculty, tailoring the sessions to specific academic disciplines (e.g., humanities, social sciences, natural sciences, and engineering). The training should cover the fundamental principles of GenAI, ethical guidelines, tool operation skills, and typical teaching application scenarios. A combination of theoretical analysis, case discussions, and practical exercises should be used to help teachers establish clear and actionable cognitive frameworks. Teachers who complete the training could receive certification, which could be included in the continuing education requirements for faculty promotions, providing institutional incentives for participation.

Additionally, it is recommended that departments or schools encourage the creation of peer support networks for GenAI teaching applications through research and teaching groups. These groups can foster a supportive learning environment by sharing experiences, presenting cases, and exploring collaborative solutions, helping teachers build successful use cases in their teaching practice, which further enhances their technical self-efficacy.

### 8.2 Construct a “performance—interest” dual-drive incentive mechanism

The study results show that both performance expectancy and hedonic motivation play a core driving role in different configurational paths, indicating that university teachers' adoption of GenAI teaching applications is influenced by both instrumental value assessments and emotional experiences during use. Therefore, universities should design incentive mechanisms that simultaneously focus on “external performance rewards” and “internal interest stimulation,” constructing a dual-drive incentive system.

At the performance level, universities should establish clear, quantifiable reward systems to encourage teachers to integrate GenAI into the development of smart courses and actively apply for “typical AI education application scenario cases.” Relevant outcomes could be categorized at the national, provincial, and university levels, with corresponding evaluation scores, and incorporated into teaching performance assessments as an important reference for faculty promotions and performance distribution. By strengthening the connection between GenAI teaching applications and teachers' career development, universities can increase teachers' recognition of the performance value of GenAI.

At the interest level, the faculty development center can regularly host GenAI creative teaching application salons or workshops, encouraging teachers to share innovative practices and teaching inspirations discovered while exploring GenAI features. Additionally, honors such as “GenAI Star Explorers” can be established, with corresponding material or developmental rewards, to acknowledge teachers' exploratory spirit in teaching innovation. By fostering an open, supportive, and sharing atmosphere, universities can enhance teachers' positive experiences and intrinsic motivation during their use of GenAI.

### 8.3 Optimize the human-AI collaborative environment to improve teaching interaction quality

The study further indicates that, in some contexts, interactivity and hedonic motivation alone can become key drivers of university teachers' adoption of GenAI. This suggests that universities should prioritize the design and optimization of human-AI interaction experiences when promoting GenAI in teaching.

To this end, it is recommended that universities deploy AI platforms tailored to local contexts to better meet specific teaching needs. On one hand, universities could collaborate with technology teams to develop specialized teaching assistants for different disciplines, such as “English Sentence Parsing Assistant” or “Mechanical Drawing Course AI Assistant,” focusing on optimizing core features such as natural language interaction, image and text content generation, and real-time feedback to ensure the practical usability and relevance of the AI in teaching. On the other hand, universities could encourage faculty and students to participate in the design and optimization of AI teaching assistants through initiatives like “Teaching AI Innovation Competitions,” fostering collaborative creation. Outstanding results from these competitions could be incorporated into the university's “AI Education Application Scenario Case” repository, promoting the continued accumulation and dissemination of best practices.

By continuously optimizing the interaction quality of AI assistants and lowering the usage barriers, universities can facilitate the transformation of GenAI from a mere “tool” into a “teaching partner,” better supporting teachers' cognitive decision-making and emotional experiences in teaching practice, thus promoting the healthy development of human-AI collaborative teaching.

## Data Availability

The original contributions presented in the study are included in the article/supplementary material, further inquiries can be directed to the corresponding author.
